# Formal education and nurses' attitudes towards alcohol and alcoholism in a Brazilian sample

**DOI:** 10.1590/S1516-31802005000400004

**Published:** 2005-07-07

**Authors:** Sandra Cristina Pillon, Ronaldo Ramos Laranjeira

**Keywords:** Attitude, Nurses, Nursing, Alcoholism, Education, Atitudes, Enfermeiros, Enfermagem, Álcool, Educação

## Abstract

**CONTEXT AND OBJECTIVE:**

Nurses are one of the largest groups of healthcare professionals sharing in patient care responsibilities, including caring for those who use and abuse psychoactive substances. The objective was to evaluate the theoretical-practical knowledge acquired by nurses in undergraduate and postgraduate studies and their perceptions about alcohol users.

**DESIGN AND SETTING:**

Quantitative, descriptive survey at Universidade Federal de São Paulo — Escola Paulista de Medicina and Hospital São Paulo.

**METHODS:**

The sample included nurses, students and nursing teachers. The survey included questions about sociodemographic characteristics; a nurses' attitudes and beliefs scale; and a questionnaire to identify formal nursing education on the use of alcohol and its consequences.

**RESULTS:**

59.7% out of 319 volunteers were nurses, 22.7% were nursing teachers and 17.6% were nursing students. 70% of the participants had received little or no information on physical, family and social problems related to alcohol use; 87% had received little or no information on high risk related to specific segments of the population; 95% had received little or no information on nursing procedures for alcohol-abuse patients.

**CONCLUSION:**

Formal education regarding the use of alcohol and its consequences is limited, especially with regard to offering adequate care and management for patients who have problems with or are addicted to alcohol.

## RESUMO

**CONTEXTO E OBJETIVO:**

Os enfermeiros constituem um dos maiores grupos de profissionais de saúde que compartilham a responsabilidade pela assistência de enfermagem prestada aos pacientes de modo geral, incluindo-se os usuários de substâncias psicoativas. O objetivo foi avaliar os conteúdos teóricos e práticos adquiridos na graduação e pós-graduação pelos enfermeiros e suas percepções sobre os alcoolistas.

**TIPO DE ESTUDO E LOCAL:**

Trata-se de um estudo quantitativo, descritivo, realizado na Universidade Federal de São Paulo — Escola Paulista de Medicina (Unifesp-EPM) e Hospital São Paulo.

**METODOS:**

A amostra foi composta por enfermeiros, estudantes e docentes de enfermagem. O instrumento para a coleta de dados foi composto por informações sócio-demográficas; Escala de Atitudes e Crenças dos Enfermeiros e um questionário para identificar os conteúdos sobre o uso do álcool e conseqüências durante a formação educacional.

**RESULTADOS:**

86% (n = 319) sujeitos participaram do estudo (59,7% enfermeiros assistenciais, 22,7% docentes de enfermagem e 17,6% estudantes de enfermagem). 70% dos participantes receberam poucas ou nenhuma informação a respeito dos problemas orgânicos, familiares e sociais relacionados ao álcool; 87% receberam pouca ou nenhuma informação sobre as populações de risco ou específicas e 95% receberam pouca ou nenhuma informação referente às intervenções de enfermagem aos pacientes alcoolistas.

**CONCLUSÃO:**

A educação formal sobre o uso do álcool e suas conseqüências apresenta limitações, principalmente no âmbito da assistência, em oferecer cuidados adequados e nos manejos dos pacientes com problemas ou dependentes de álcool.

## INTRODUCTION

Two fields of study are revealed through reviewing the literature^1-3^ that attempts to understand attitudes and beliefs surrounding psychoactive substance use and addiction. The first involves the identification of attitudes, points of view and perceptions among healthcare professionals in relation to this problem. The second investigates the reasons that lead to a lack of awareness or understanding among these professionals regarding substance use.

Nurses are one of the largest groups of healthcare professionals sharing in patient care responsibilities in general, including caring for those who use and abuse psychoactive substances. They may encounter users in various sectors of the healthcare system. It can therefore be assumed that nurses should be able to assess their own attitudes relating to alcoholism, so that they can care for patients without making value judgements, as demonstrated in studies^1-3^ presented previously.

There is evidence that negative attitudes exist among nurses in relation to patients who are psychoactive substance users. Recognition of inappropriate attitudes and reflection on behavior constitutes the beginning of a process to modify the nurse's behavior. The process of evaluating attitudes includes recognition of the origin of such attitudes, investigation of how these affect the care given to patients and seeking of knowledge through formal education. This latter provides a scientific basis and can be influential in changes.^3^

It is essential to evaluate beliefs and values that affect the care given to others. Thus, the inclusion of conscious evaluation is fundamental for the professional nurse. However, little is known about the attitudes (thoughts, feelings and behavior) of nurses in relation to patients who are drug or alcohol users. In addition to the lack of formal research, there has been very little training (continued education), or even formal education on this theme included in undergraduate nursing courses.^1^ Nevertheless, this information is considered indispensable in preparing nurses to offer qualified care for this population.

The limited educational background of nurses contributes greatly to the formation of attitudes and value judgements. This fact is reflected in surveys.^4^ These show that, when dealing with patients who are alcohol users, nurses' attitudes and beliefs are significantly more negative and permeated with moral content than when dealing with other patients.^4^ Neglect in identification and early intervention may aggravate alcohol users' problems and may render their prognosis more serious, to the point of the condition becoming chronic.^4^

### The need for teaching about alcohol use in nursing courses

Although health organizations officially recognized alcoholism as a disease in the 1950's, it was much later that this was accepted by the population in general. Many nurses and other professionals have been reluctant to care for such patients. Specifically, there has been reluctance to acknowledge problems related to alcohol use and abuse and thus to make the necessary interventions and referrals.^2,5^

The harm to individual and family health, as well as social problems associated with the use of alcohol and drugs, began to generate research proposals related to the education of nurses, because there had been little attention given to this matter in nursing school curricula.^1^

The increasing needs of patients with alcohol-related problems require that nurses should be educated in this field in order to be able to respond to such needs. This provides an incentive for seeking new institutional and social approaches, thus placing nurses at the forefront of discussions regarding practical care, education and research. Nursing practice for alcohol-related problems depends on constantly changing social influences. The knowledge of the current social situation influences the agents (in this case the nurse and the substance user) and is influenced by the agents, in a dialectical relationship. The nurse's actions influence the alcohol user in the same way that the alcohol user's actions have some influence on the approach used by the nurse.

The nurse's performance may be an essential resource in assisting individuals, families and communities that have problems related to the use of psychoactive substances. However, to be an effective resource requires practical, cognitive and relational abilities that have already been developed, or an aptitude for these.^1^

Changes in the population's healthcare needs and continuing high rates of alcohol use demand compatible healthcare services or programs. Practicing nurses should be able to absorb such changes. Demands on the healthcare system related to the use of psychoactive substances are requiring healthcare professionals, including nurses, to have knowledge of such matters.

Nurses have the potential to respond to this challenge if they explore new alternatives, adapt their nursing care plans in general and provide assistance to patients with problems due to alcohol use.^3^

The influence of formal education on nurses' attitudes in situations involving alcohol and drug abusers needs to be studied in a more structured manner. If formal education were indeed a significant determining factor, the repercussions might be very serious. Successive graduating classes from many schools would, if they did not seek additional education, continue reproducing the same inadequate attitudes when carrying out their nursing practice.

In Brazil, there have been very few studies on nurses' attitudes towards alcoholism.^6^ This makes such investigations even more important. Our aim was to evaluate the theo- retical/practical content of undergraduate and postgraduate nursing courses and the attitudes of graduates from these programs when working with alcohol abusers.

## METHODS

A quantitative, descriptive study on nurses' attitudes (thoughts, feelings and behavior) when dealing with alcohol abuse patients was conducted. This study was the continuation of an investigation done as part of a master's degree dissertation, which began in 1998.^7^

### Sampling and location

The sample consisted of 370 participants. Of these, 60 were fourth-year nursing students from the Nursing Department at Universidade Federal de São Paulo – Escola Paulista de Medicina (Unifesp-EPM); 220 were nurses at Hospital São Paulo and Sociedade Paulista para o Desenvolvimento da Medicina; and 90 were teachers in the Department of Nursing at Unifesp-EPM.

### Measurements

A questionnaire was developed to assess 1) personal information on participants (i.e. age, sex, time since graduation, time in profession and professional qualifications); and 2) their assessment regarding alcohol use and its consequences, as acquired during undergraduate and postgraduate studies. Participants were requested to mark the options according to their knowledge and when such information was acquired (undergraduate, specialization, masters' and doctorate levels). This part of the questionnaire contained 18 items that they were asked to evaluate. These items were divided into three groups. These allowed for the identification of material presented, regarding problems with alcohol use (e.g. physical and mental health, and social problems) in specific segments of the population, or among those at high risk from alcohol use (i.e. adolescents, the elderly, and pregnant women). This section also included questions on the nursing care or assistance that can be offered to alcohol abuse patients. The "Beliefs and Attitudes of Nurses in relation to Alcoholism" scale from the NEADA Project (Nursing Education in Alcohol and Drug Education)^8^ was utilized. This scale became part of the alcohol and drug training program for nurses, students and nursing teachers in Connecticut, United States, in 1985. It should be noted that the present study was the first to use this scale in Brazil.

The translation into Portuguese and back translation of the questionnaire for the scale was done by two professionals: one Brazilian (Prof. Ronaldo Laranjeira, PhD) and one Englishman (Prof. John Dunn, PhD), both of whom are specialists in substance addiction. The inter-item reliability was evaluated using Cronbach's alpha. The inter-item agreement for the 30 items among the 319 nurses was 0.90. The result was considered adequate for use.

The scale is composed of variables that were developed through professional experience in working with alcohol abuse patients. This demonstrates that the scale had initial face validity. The purpose of the scale is to evaluate the nurses' understanding of alcohol use, by using a total of 30 responses on a Likert-like scale that was divided into 5 subscales.

The components of the subscales are:

- Nurses' understanding of theoretical concepts relating to alcohol use and dependence;
- Nurses' understanding of alcohol abuse patients' privacy needs;
- Nurses' understanding of their professional and personal feelings towards working with such patients;
- Nurses' understanding of their professional preparation for working with such patients; and
- Nurses' understanding of nursing procedures when caring for such patients.

In order to standardize the answers, a "Likert scale" style was chosen, with answers ranging from 1 (strongly disagree) to 5 (strongly agree). These types of answer verify the participant's level of agreement or disagreement with a series of affirmations that express something favorable or unfavorable in relation to what is being studied.

Data were collected over a two-month period (September and October 1998), from volunteers recruited through personal contact, workplace contact (i.e. nurses and teachers), and association with fellow students in classes or internship situations. The questionnaires were distributed and then returned within 24 hours.

Prior to data collection, approval was given by the "*in Anima Nobili*" Clinical Research Ethics Committee of Universidade Federal de São Paulo – Escola Paulista de Medicina, and the Nursing Board of the hospital (Resolution 96/196). Participants were informed of the purpose of the survey and confidentiality was guaranteed. A pilot study was done (n = 10), in order to evaluate any difficulties that might arise in understanding the questionnaire.

A database was developed that was compatible with the Statistical Package for the Social Sciences (SPSS), version 8 for Windows, thus enabling descriptive and comparative analysis. The sociodemographic characteristics of the population were described using measurements of central trends and variability. Kruskal-Wallis non-parametric analysis was used for evaluating several independent samples, through comparing two or more groups (e.g. nurses, students and teachers) with regard to all variables (e.g. alcohol use and its physical, family and social consequences; and specific or high-risk segments of the population). In all cases, a 0.005% risk of committing a type 1 error was adopted.

## RESULTS

Of the 370 subjects who received the survey, 319 (86%) responded. The remainder of the forms (14%) were not returned or were returned blank. The justification given by some nurses for not completing the questionnaire was a lack of time.

Among those that responded, there were 190 nurses (59.6%), 74 nursing teachers (23.1%) and 55 nursing undergraduates (17.2%), all in their fourth year of nursing education.

Of the 319 who responded, 94% (300) were women. The students' average age was 23.73 years (standard deviation, SD = 2.75 years) and the range was 20 to 37 years. For nurses, the average age was 31.65 years (SD = 6.65 years) and the range was 22 to 53 years. For nursing teachers the average age was 40.38 years (SD = 7.96 years) and the range was 28 to 60 years. Of those surveyed, 66% of the nurses and 86% of the teachers were graduates from public universities.

With regard to the teachers' educational qualifications, 45% either had obtained or were working towards their doctorate, 82% either had a master's degree or were working towards it, and 88% had obtained a specialization qualification in some field. Among the nurses, 7% had obtained a master's degree or were working towards it, and 55% had specialist training in some field or were taking courses. Thirty-eight percent of the nurses had only done undergraduate studies. Of the student nurses, 100 percent were in their fourth year of nursing education.

Ninety percent of those surveyed had received either a little or a lot of information during their formal schooling (undergraduate or postgraduate) regarding alcohol-related problems (health, family and social). Eighty- four percent of those surveyed had received a little or a lot of information regarding specific segments or high-risk segments of the population in relation to alcohol use (adolescents, the elderly, pregnant women and others).

There were evident limitations in the teaching on alcohol and related subjects in nursing programs. Nurses had received knowledge that in some way allowed them to identify problems resulting from alcohol use and to identify individuals in high-risk segments of the population. However, they had received very little information on the nursing care for this group. The data showed that the material was taught, but there was no way of evaluating the quality of what was taught, or how much was taught when the nurse was still a student.

There was a significant relationship between the quantity of information received about alcohol use and its consequences (physical, family and social), and the nurses' understanding of relevant nursing procedures (H = 10,222; p < 0.005), i.e. nursing care for alcohol abuse patients.

There was also a significant relationship between knowledge about alcohol use in specific or high-risk segments of the population (adolescents, the elderly, pregnant women and others) and nurses', students' and nursing teachers' understanding of alcohol abuse patients when preparing to working with them. In other words, lack of knowledge about specific or high-risk segments of the population affected competence (H = 18,621; p = 0.000).

There was no significant relationship between the education received regarding nursing procedures for alcohol abuse patients and the attitudes among the nurses in the three groups regarding alcohol abusers' feelings and privacy, and the concepts of alcohol use.

Comparison of the quantity of information acquired by the subjects showed that there were significant differences between the nurses, students and nursing teachers regarding their understanding of procedures used in the nursing care of alcohol abuse patients (H = 17,460, p < 0.000).

## DISCUSSION

On the question of what had been taught about the physical, family and social problems resulting from alcoholism, more than half of those surveyed indicated that they had only received a minimum amount of education. There are very few studies that have considered this matter. One study, conducted in the United States,^9^ investigated the general subjects that touched on alcohol and drug use within a nursing program, and the extent of such education among nurses in a general hospital, before they did specific training. In contrast to the data from the present study, this author found that 16% of the nurses had never had any teaching on this matter and, among the rest, 28% reported receiving very little teaching, and 31% little or very little teaching. This author's findings were in agreement with the results from another hospital survey in the United States^10^ that indicated that the information offered to nurses about alcohol and drugs was relatively insufficient.

After evaluating our results on the education provided to nurses regarding alcohol users, we observed that only a minority of the participants had received appropriate education. These findings, together with others,^11,12^ lead to the conclusion that nurses are still resistant to the inclusion of education that would assist them in helping alcohol users with their daily responsibilities. As a consequence, the offer of assistance directed towards alcohol abusers' problems still falls short of their healthcare needs.

In general, well-documented course content in nursing curricula regarding alcohol and drugs would depend on good definition. As demonstrated in a survey,^12^ the subjects offered in most courses allot very few hours for teaching related to alcohol and drug use. Experience has shown that, in most cases, such teaching is done by a professional who is not a specialist in that field. In a more specific analysis of the problem, the above survey^12^ revealed that the majority of nurses, perhaps as a result of their interest and/or need for theoretical-practical knowledge, had acquired basic knowledge about alcohol use and its consequences.

It can be seen that the results from the present study do not differ from those of other studies that indicate that nurses have limited knowledge in this field. Although the nurses, students and nursing teachers had received instruction regarding theoretical aspects of alcohol and its consequences, no timely initial interventions were taking place in practice. Perhaps this was because of an unwillingness to take on a commitment towards including those activities in their daily jobs. Nonetheless, such early identification of these patients would facilitate onward referral to specialists.

**Table 1:** Sociodemographic data on nurses (n = 319) enrolled in a study about their attitudes and perceptions towards alcoholism

	Nursing students		Nurses		Nursing teachers	
	n	%	n	%	n	%
	55	17.2	190	23.2	74	59.6
**Sex**						
Female	53	96	175	92	72	97
Male	2	4	15	8	2	3
**Age**						
	Mean: 23.73 years		Mean: 31.65 years		Mean: 40.38 years	
	SD: 2.75 years		SD: 6.65 years		SD: 7.96 years	
	Min: 20 years		Min: 22 years		Min: 28 years	
	Max: 37 years		Max: 53 years		Max: 60 years	

*SD = standard deviation; min = minimum; max = maximum.*

With regard to nursing procedures, the approach towards such patients and the specific care, which is the essence of a nurse's work, are not included in the material taught. There is not even the material considered necessary for ensuring a minimum of care. This is definitely a cause for concern and requires special attention from educational institutions. It is important to note that the aim here is not to direct knowledge of alcohol and other drugs towards a specialization but to focus on the fact that each and every professional has a responsibility to address the issue of alcohol and other drug use. Thus, professional nurses have a responsibility to recognize the levels of complexity of the situations.

There is a conviction that alcohol-related problems should only be dealt with by specialized nurses. Other nurses can and should participate in caring for such patients, since they may be present in every sector of the healthcare service. The separation of these patients is not justified. Ninety percent of nurses in the present survey received basic instruction and 80 percent received more advanced instruction regarding the problems of alcohol use. In theory, they have some capacity to give assistance.

For nurses to resolve contradictions of this type and assess the care that they give to such patients and their families, information focusing on specific problems is needed. Nurses will benefit from support and supervision given by specialized nurses who have a reputation in this field as a result of proven quality in their professional activities. Other trained professionals may also be of assistance.

It was found that 19 and 10% of nurses and nursing students respectively had received no teaching. However, since most of them did acquire the information, we assume that there was an incentive to gain knowledge. This could be considered to be an initial effort on the part of nurses, students and nursing teachers to become knowledgeable in this area.

For the course content relating to nursing care for alcohol abuse patients, the need to reinforce and invest in formal education should be taken into consideration. Regularly offering theory and training resources that go beyond the identification of the problem, and include possible approaches towards sensitizing (minimum procedures), treating (detoxification) and psychosocial rehabilitation (training in social skills), are needed. Care could be offered before referral to specialized services and would be at the first level of complexity suggested in the United Kingdom Advisory Council model.^11^

In this model, preparation for working with alcohol and drugs at various levels of complexity is recommended. Basic information would be given to nurses that work in general services, and intermediate information would be available for those working in specialized areas where there is a greater frequency of alcohol and drug-related problems (psychiatry, gastroenterology and emergency). An advanced level of information would be offered to those dedicated to working with those addicted to alcohol and other drugs.

Nurses have the potential to develop care that is optimal for patients with alcohol-related problems, because of the frequency with which they come up against these problems in all healthcare sectors. Therefore, most nurses will, in addition to providing general care, have some opportunity to inform, counsel and give clarifications regarding the use of alcohol and its harm to health.^13^ Some patients may feel more at ease speaking to a nurse than to another professional, since the nurse would perhaps be more available because of the length of time for which these patients require nursing care.

In units specializing in the treatment of alcoholics, nursing activities include traditional responsibilities such as administering medications, observing and registering clinical symptoms (e.g. withdrawal symptom severity, pulse and blood pressure) and the planning and provision of 24-hour care. At the same time, as in most other fields of psychiatric nursing, these traditional functions are only one part of the activities carried out by nurses, because dealing with such patients requires specific abilities for the kinds of procedures used. Most therapeutic procedures may be performed by nurses, such as instructing the patients regarding education and health, counseling, motivating them to follow through on medical treatment, evaluating their physical and mental state, informing them about laboratory tests, giving clarifications, guiding their families or providing follow-up to avoid recurrences. These activities are part of the treatment for alcoholism and may be carried out in the community or in a home atmosphere. However, some of these must be carried out by a nurse with advanced skills and preparation for caring for alcohol abuse patients.

Nurses are very often part of the community health teams and are involved in the treatment of alcoholism. They may work in hospital-related activities in hospital units, or in non-medical institutions where alcohol abuse patients are treated. These may include therapeutic communities. For this reason, nursing schools need to invest time in training students in matters related to alcohol and other drugs.

### The influence of knowledge regarding alcohol on nurses' understanding

Investigative studies^1-3,14,15^ have considered that the acquisition of specific knowledge brings changes in unfavorable or negative attitudes towards alcohol abuse patients and, in consequence, influences the quality of care provided.^10^ In the present study, the relationship between the amount of information on "alcohol and its consequences (physical, family and social)" and "specific segments of the population that are at high risk from alcohol use" that had been acquired by nurses, students and nursing teachers was significantly different between the three groups. Students had received less information than had nurses and teachers. This suggests that knowledge may be acquired after graduation, since professional life, both in schools and medical services, allows for greater possibilities for improvement in areas of theory and practice, and also provides practical contact with the problem. However, regarding the amount of information received on "nursing care for alcohol abuse patients", there was no evidence of difference between the groups surveyed: they all uniformly lacked information. In their assessments of the amount of information they had received regarding "teaching on the use of alcohol and its consequences (physical, family and social)", there was a difference only with regard to procedures. This may suggest that the quantity of information acquired on the subject can influence their understanding of procedures while providing nursing care for alcohol abuse patients.

**Table 2:** Comparison between attitudes towards alcoholism and quantity of information acquired by nurses, students and nursing teachers (n = 319)

**Alcohol use and its consequences (physical, family and social)**	**Interventions**
	H = 10,222; p < 0.05
**Specific or high-risk segments of the population**	**Education**
	H = 18,621; p = 0.00
**Nurses versus students versus nursing teachers**	**Privacy**
	H = 17,460; p = 0.000
	**Procedures**
	H = 10,359; p = 0.00

*Kruskal-Wallis test significance level: p < 005.*

This may be related to nurses' emphasis on general patient care, with no difference in the care given to alcohol abuse patients. Nurses may consider that nursing care should be offered to all patients in a generalized manner, with no distinction made. Because alcoholics are only seen as people who have a particular illness, it is possible that nurses do not realize the need for directing specific care towards them, related to their alcoholism. Also, since chemical dependence is a new area in nursing, it may not yet have aroused nurses' interest. It should be noted that, since education regarding these problems is part of the psychiatric curriculum, an area of nursing that is not highly sought among nurses, general nurses would be very unlikely to seek more knowledge about patients who can be "evaluated and referred" to specific medical services. However, these conjectures need to be scrutinized in carefully thought-out research studies.

Travelbee^16^ stated that professionals with little or no teaching on alcohol use and its consequences have little to contribute towards nursing care for this segment of the population. Her study is considered a landmark in the literature on nurse-patient interpersonal relationships. She declared that the nurse's understanding of the patient is the factor that determines the quality and quantity of nursing care that may be offered.

With regard to the quantity of information acquired on "specific or at-risk segments of the population (adolescents, the elderly, pregnant women and others)", the results from the present study showed that education was the only significant point among the nurses' answers (students, nurses and nursing teachers). This suggests that the amount of knowledge received about vulnerable segments of the population was insufficient.

This finding is a matter for concern because knowledge of segments of the population that are at risk from alcohol use or abuse can diminish nurses' feelings of fear, anxiety and concern regarding how they are performing their professional activities. The nurses in the study by Farinazzo and Beraldo experienced all of these.^17^ Travelbee reported that good professional education assures and promotes good quality of life at work.^16^ In our study, there were no significant differences in the perceptions of the students, nurses and nursing teachers surveyed with regard to lack of information received about segments of the population that are at risk from alcohol use or abuse. This finding is in agreement with studies in other countries that have indicated limitations in nurses' educational preparation (theoretical knowledge and practical abilities) relating to alcohol and drugs.^18,19^ An investigation in Brazil^12^ showed this same lack of education in the curricula of 24 undergraduate nursing schools.

These findings testify once again to the need to make educators in all nursing programs aware of the necessity for greater investment in teaching nursing students about alcohol and other psychoactive substances. The finding also shows the value of giving incentives to teachers to acquire a minimum of specific knowledge about this topic.

In evaluating the differences in perceptions among nurses, students and nursing teachers, it was seen that nurses were more aware of the procedures used in nursing care than were students and nursing teachers. The difference in perceptions may be related to the diversity of patients with alcohol-related health problems that they care for in practical clinical situations. Such problems had culminated in the need for hospital assistance. Perhaps nursing teachers and students do not have as much contact with alcoholics. The results showed that nurses do not acquire sufficient information on this matter, but more than half are specialized in some field. Perhaps the diversity of clinical care causes them to constantly seek training, formal or otherwise, for the acquisition of practical- theoretical support, so that they can provide care for alcoholics.

In the light of this professional motivation, there should be more courses for updates on alcohol and drugs. In the present study, the understanding of alcohol use among the three categories of nurses was significantly different. The nurses and students had a very similar view of patient privacy, but differing from the teachers' perception. For the teachers, questioning the patient on this matter could constitute an invasion of privacy, and this concern was much greater than among nurses and students. This finding should be taken into consideration, since the teachers are in an educationally much better informed category (p < 0.0001). Therefore, knowledge in itself does not seem to guarantee a realistic perception. Some people may find this notion uncomfortable.

## CONCLUSION

The void in this field of nursing education may be contributing towards neutral or negative attitudes. This may, in turn, be jeopardizing the care offered to the segment of the patient population who use or abuse alcohol or other psychoactive substances. Formal education regarding the use of alcohol and its consequences is limited, especially within the sphere of offering adequate care and management for patients with problems of alcohol or alcohol addiction. It is imperative that nurses should be able to identify problems related to alcoholism, when they appear together with other health problems, so that they can have the capacity to care for such patients.
